# Mr. Webster’s Brain

**Published:** 1853-01

**Authors:** 


### MR. WEBSTER'S BRAIN.

Dr. Jeffries, Mr. Webster's medical attendant, has given, in the January number of the *American Journal of the Medical Sciences*, an account of the last illness of the great statesman, which we propose to copy in our next number. In the mean time we give the admeasurements of his brain, as taken by Dr. Wyman, who was present at the post mortem exiamnation.

Cuvier's brain is the largest of which we have any account in works on anatomy, and weighed 4 lbs., 5 drs., and 10 grs., or 28,147 grains. Mr. Webster's weighed 3 lbs., 15 ozs., 12 drs., or 27,891 grs—just 256 grains less than that of the renowned naturalist. Dupuytren is mentioned in several physiological works as having had a brain only a little less than that of Cuvier; but it turns out to have been inferior in size to that of Spurzheim and Abercrombie, and to have weighed 14 ozs. less than Mr. Webster's. The circumference of Napoleon's head is stated to have been 23 inches; that of Mr. Webster's was 23¾ inches. From ear to ear over the top of Cuvier's head was 15 inches; Mr. Webster's measured the same in that direction. The capacity of his cranium was 122 cubic inches, while of the 623 crania measured by Dr. Morton, the capacity of the largest was only 114 cubic inches. That of Mr. Webster exceded any which has yet been recorded. The brain evinced marks of disease, and it was remembered after his death that he had manifested a short time previously some hesitation of speech, and other slight indications of nervous disorder.

